# Existential loneliness and life suffering in being a suicide survivor: a reflective lifeworld research study

**DOI:** 10.1080/17482631.2022.2122157

**Published:** 2022-09-08

**Authors:** Christina Nilsson, Karin Blomberg, Anders Bremer

**Affiliations:** a Faculty of Medicine and Health, School of Health Sciences, Örebro University, Örebro, Sweden; b Division of Emergency Medical Services and University Health Care Research Center, Örebro University Hospital, Örebro, Sweden; c Faculty of Health and Life Sciences, Department of Health and Caring Sciences, Linnaeus University, Växjö, Sweden

**Keywords:** Existential loneliness, lived experience, phenomenology, reflective lifeworld research, stigma, suicide, suicide survivors, suffering

## Abstract

**Purpose:**

The aim of the study was to describe the loss of a family member by suicide, based on the lived experience of suicide survivors.

**Methods:**

A phenomenology study with a Reflective Lifeworld Research approach was conducted, consisting of sixteen interviews with eight suicide survivors.

**Results:**

The essence of losing a family member by suicide encompasses experiences of involuntary and existential loneliness, life suffering, and additional burdens in a life that is radically transformed, comprising prolonged and energy-intensive attempts to understand. Life for the family member encompasses a constant fear of being judged and an ambiguous silence, where this silence can both lead to involuntary loneliness and be a source of support and fellowship. Support mechanisms inside the family fall apart, and it becomes obvious that the survivors’ experiences affect others. The loss also implies an active endeavour to maintain the memory of the deceased.

**Conclusions:**

Based on these results, it is important for professionals to accept the survivors as suffering human beings early—from the point of the notification of death—and consider them as patients in need of compassionate care. Such support might reduce life suffering, counteract stigma and involuntary loneliness, and work simultaneously as suicide prevention.

## Background

A sudden death is unexpected and unforeseen, and happens without warning when compared to an expected death, which happens gradually and allows the family to prepare for the loss. Suicide is often considered a sudden death and situates the suicide survivors within a major life transition (Iserson, 2000). Every year, more than 700000 people die due to suicide, which means that one person dies every 40 seconds (World Health Organization, 2022). For each suicide, an average of about 15 extended family members are estimated to be affected (Berman, 2011). This means that 11500000 suicide survivors are left behind every year.

In previous studies, both quantitative and qualitative differences have been found between the loss of a loved one by suicide and other types of bereavement. Suicide survivors are at risk of a range of adverse mental and physical health outcomes compared with the general population (Erlangsen et al., 2017). A family who has been affected by suicide has a higher risk of being affected by suicide again (Niederkrotenthaler et al., 2012; Runeson & Asberg, 2003; Tidemalm et al., 2011; Qin et al., 2002). However, quantitative methods may fail to identify differences in bereavement when suicide-specific domains are not considered, for example, meaning-making around the death; feelings of guilt, blame, and responsibility; and feelings of rejection and abandonment (Jordan, 2001). A systematic literature review indicates that suicide is different and more difficult when compared with bereavement after different types of deaths (Jordan, 2001). Suicide survivors are not only faced with the typical reactions that occur following a death, but they also have unique experiences, such as the unending question, “*why?*” (Pompili et al., 2013). Another systematic literature review has also found that suicide survivors’ grief differed significantly concerning levels of rejection, shame, stigma, blaming, and concealing the cause of death when compared with all other survivor groups (Sveen & Walby, 2008). Previous research has focused on the bereavement process following suicide (Shields et al., 2017), but has not fully addressed the essence of what it means to lose someone by suicide by openly and curiously engaging with the suicide survivors’ own stories. By telling their own stories, the suicide survivors are given the opportunity to address their experiences honestly and in an uncensored way to convey what the loss by suicide means to them. Such stories make it possible to understand the essence of this phenomenon. Therefore, the aim of this study was to describe the loss of a family member by suicide, based on the lived experiences of suicide survivors.

## Research design

This phenomenological study was based on the Reflective Lifeworld Research (RLR) approach, where the ontology is drawn from Husserl (1936/1970) and Merleau-Ponty’s (1945/2002) lifeworld theory. RLR advocates that researchers must “go to the things themselves” and discover human experience in all its variety in order to obtain a profound understanding of the phenomenon (Dahlberg et al., 2008). The phenomenon in this study is losing a family member by suicide.

### Recruitment of participants

The inclusion criteria for participation in the study were: a) confirmed suicide outside hospital; b) suicide within the last three years; c) death notification provided by a professional with a duty to perform this task; and d) suicide survivors residing in a specific medium-sized region (36 inhabitants/km^2^) in Sweden. In comparison with other countries, Sweden is described as a society based on secular-rational and self-expression values, which means that less emphasis is placed on religion, traditional family values, and authority. Divorce, abortion, euthanasia, and suicide are seen as relatively acceptable. Further, a society with self-expression values gives high priority to environmental protection, growing tolerance of other cultures, LGBTQ+, and promoting gender equality (World Values Survey, 2022). The exclusion criteria were: suicide survivors who the first author had met in her work as a prehospital emergency care nurse. Participants were recruited by publishing an advertisement in two daily newspapers and by posting advertisements in seven grocery stores located across a wide geographical spread in the region. People were instructed to send an email or call to a toll-free number to report their interest in participating. Thereafter, an information letter was sent to those who indicated an interest in participating in the study. A total of 14 suicide survivors responded to the advertisement. Five persons did not meet the inclusion criteria, as the cause of death was not confirmed as suicide, the death happened inside a hospital, or they had received the death notification from a family member. One potential participant declined the invitation to participate. The eight participants who were included were composed of five women and three men, aged between 42 and 74 years. The time since the loss of the deceased ranged from three months to up to three years before the first interview. The re-interviews were conducted six months after the first interview. The deceased were six men and two women, and the type of relation to the person who died by suicide included brother, daughter, husband, mother, partner, and son, and there were different types of suicide. Seven of the participants received the death notification from a police officer, and one from a general practitioner.

### Data gathering

Individual interviews were performed with eight suicide survivors in the first round, with a second re-interview held between three to six months afterwards, which resulted in a total of 16 interviews. The first author performed all of the interviews, which took place from September 2019 to March 2020. The interviews in the first round proceeded by posing an initial question: “*You lost your X (relation) by suicide, can you please tell me about it?*” To obtain a deeper understanding of the phenomenon, the participants were encouraged by asking further questions and probes, for example: “*Can you describe a little bit more about … ?*”; and “*Can you tell me more about what you mean by … ?*”. Three pilot interviews were completed with suicide survivors to test the interview approach and the initial question. The pilot interviews did not lead to any changes in the questions and were not included in the analysis.

The first round of interviews lasted between 41 and 89 minutes (mean 63 minutes and median 64 minutes) and, after the initial question, the survivors spoke for a median of 12 minutes (mean 15 minutes) before other probes were given. After the first round of interviews, a close reading of all the transcripts was made, aiming to identify and clarify any unclear meanings in the data, so that the authors were able to grasp the participants’ own words and descriptions to avoid making any assumptions or misunderstanding their meanings. In the re-interviews, two questions were first posed to each participant: *“Is there something that you have thought about after the first interview?”* and *“Have you something further that you want to tell me about since the last interview?”* Additional questions, based on the readings of the interview transcripts from the first round, were prepared for each participant. The interviewer asked for clarification about responses given during the first interview, and the participant was given the opportunity to further elaborate on their previous descriptions and to clarify using other words. The re-interviews lasted between 37 and 113 minutes (mean 71 minutes and median 64 minutes). The participants chose the time and place for the interviews. The first round of interviews were held in either the participants’ homes (*n* = 3) or in different care units (*n* = 5). The re-interviews were also held either in their homes (*n* = 4) or in different care units (*n* = 4). If needed, the moral obligation to protect the participant’s health was temporarily given first priority, and the research requirement became secondary if the participants presented suicidal behaviour.

### Data analysis

According to Gadamer (1960/2013), our preunderstanding is a prerequisite for understanding—to maintain a critical attitude as researcher is crucial. The willingness to understand the interviewee and the methodological principles in RLR consist of openness, curiosity, and bridling, and help the researcher in developing an understanding of the phenomenon (Dahlberg et al., 2008). All interviews were audio-recorded and carefully transcribed verbatim by an experienced research secretary to include non-verbal information. The tripartite structure of the data analysis comprised a movement between the whole—the parts—the whole (Dahlberg et al., 2008). The initial phase aimed to allow the researchers to become familiar with the data. The first author listened to the first-round interview and the re-interview for each participant and read the transcripts several times to get a sense of them as a whole, using a sense of curiosity and openness to open up the mind to the text and the meanings that are present. This involved looking for otherness or something new, rather than looking for things that confirm what is already known (Gadamer, 1960/2013). One of the co-authors read all of the transcripts and it was a conscious choice to allow the other co-authors to read several of the transcripts. The rationale was to allow the possibility for all to ask curious and critical questions about the analysis and the meanings so that the analysis did not proceed too quickly. Bridling is a process and an approach that involves researchers moving between subjective and objective dimensions in their ambitions to investigate and uncover meanings that are valid. All of the authors used bridling throughout the whole analysis, to “keep an eye on and keep in check”—to slow down and incorporate a continuous investigation of one’s own preunderstanding and so avoid making assumptions and jumping to conclusions (Dahlberg & Dahlberg, 2019, 2020) and, further, to be open to new understandings (Dahlberg et al., 2008). After the initial readings, keeping the phenomenon in focus, an understanding of the meaning structure presented in the data was illustrated by describing essential, nuanced, and varied meanings. Then, all authors worked together to cluster meanings that possess the same characteristics and which might be related to each other, including all the variations of the phenomenon. This clustering formed a broader understanding of patterns within the phenomenon. The analysis was performed by all of the authors and, to facilitate the clustering process within the extensive data, the NVivo software package version 12 (QSR International, 2018) was used. A new whole description, comprising the essential structure of meanings that explicate the phenomenon, was presented. First, the essential structure of meanings was written. Then, the individual constituents were formulated, including variations within meanings and unique experiences, and by presenting quotations (Dahlberg et al., 2008). To illustrate the findings and to increase the trustworthiness of the findings, quotations were identified. According to the RLR approach, the results must be presented in the present tense (Dahlberg et al., 2008).

### Ethical considerations

Several established researchers in this area have previously studied suicide survivor experiences, and each predominantly adopts a positive attitude towards the ethical implications of including such participants in studies (Andriessen et al., 2022; Dyregrov, 2004; Dyregrov et al., 2010; Hawton et al., 1998; Omerov et al., 2014; Runeson & Beskow, 1991). Andriessen et al. (2022) conclude, in a study with 61 bereaved family members, that the participants experienced little distress and would recommend participation to others. Dyregrov et al. (2010) found, in their study with 92 suicide survivors, four categories of motivations for individuals to participate in such research: “*Helping Others*”, “*Venting*”, “*Insight*”, and “*Just Because*”. Their conclusion was that the suicide survivors are motivated to participate in interviews and that the motives are multifaceted and reflexive. Further, suicide survivors should be allowed to decide for themselves whether to participate in research or not.

The researchers were fully aware that the participants could regard the topic of this study as being sensitive. Therefore, all participants received the phone number of a counsellor in the event that anyone needed further support before or after the interview. The authors prepared a plan for how to respond if a participant expressed suicidal thoughts during the interview. After the interview, the first author’s ambition was to change from the role as researcher to that of prehospital emergency care nurse and make an assessment according to medical service guidelines. Further standard procedures related to the assessment would be followed. Ethics approval was obtained from the Swedish Regional Ethical Review Board of Uppsala (No. 2017/228 and No. 2017/228 K) and the Swedish Ethical Review Authority (No. 2019/03259). The participants were informed in writing and orally that their participation was voluntary and that they had the right at any time to refrain from further participation without specifying any reasons. Before the interview, written informed consent was obtained from all participants.

## Results

### Essence

The loss of a family member by suicide is a phenomenon characterized by existential loneliness, which means life suffering with additional burdens being placed on those closest to the deceased. These include the perception that the family member does not have the strength to live any longer and experiences feelings where their existence seems to crack. Everyday life means heavy feelings of guilt and a prolonged and energy-intensive effort to try to understand. At the same time, to understand is hard to bear, as it presents painful insight into unknown elements about the deceased. Life includes existential loneliness and silence from people around them, while at the same time there is an appreciable need to speak to someone who dares to remain silent and listen. This ambiguous silence can both lead to involuntary loneliness and be a source of support and fellowship. Disclosures in relationships where someone is listening are exposed to extreme challenges and the usual support mechanisms inside the family become unstable. There is a strive to seek an existence that is familiar, despite the fact that life has been radically transformed. The loss implies an active endeavour to maintain the memory of the deceased and manage the grief to prevent a total obliteration of the deceased’s imprint. Stigma and a constant fear of being judged and held liable is constantly present in life, which also involves embarking on experiences that adversely affect others and contributes to an undesirable identity.

The following constituents further describe the phenomenon: *A loss with additional burdens; The need to understand; The ambiguous silence; Memories as an eternal companion*; and *The fear of being convicted*.

### A loss with additional burdens

Loss from a suicide constitutes more burdens of grief and produces extra “layers” of pain. To be left behind is an additional burden: first, in the sense that a family member did not have the strength to live any longer, and second, by actively ending their life. The realization that insurmountable worries led to the loss of the family member is painful:

We [the family] have probably talked a lot about that part precisely because (pause) yes, because for the debt issue as well, as one feels guilty because someone is so sad that they do not want to live. So then, yeah, so that [the insight] we probably have, that [the insight] has been the major part.

The nearest time after the family member’s death is dizzying and painful. The suicide survivor breaks down, stops and feels, but also switches off and manages under this “shutdown” to arrange practical tasks. It is like having a nightmare, where the whole world seems to be cracking. In connection with the death notification being delivered, difficulties are experienced in understand what is being said, panic takes hold, and reactions such as crying, screaming, and anger arise. The bodily feelings associated with the death notification remain as an embodied memory that can return at any moment. The death notification can be described as a complete shock, an unreal dream, a brutal message, a total change of life where reality is questioned, and at the same time is so obviously real.

You just lose everything. It is uncontrollable. It is like you just, you cannot control it. It is a sign that you are alive, to a very high degree. That’s how you can express it. That you are, you are, you cannot be more alive.

The death notification is surprising for some, but not surprising for others, and, in some cases, the notification was only a formal legal process, as they already knew that the family member was dead. It can also imply a long and lonely wait to obtain information. The sense of powerlessness was palpable when the survivor had to wait patiently for the police officers to deliver the death notification to other family members. Further burden occurs when the survivor tries to convey the deceased’s mood before death in meetings with healthcare professionals and they feel that the response is cool. The inability to provide care allows the blame to be partially shifted onto the healthcare service. At the same time, this insight into the healthcare service’s shortcomings and inadequacies burdens the survivor and they express how feelings of powerlessness, betrayal, and disappointment take hold. Further burden and pain are experienced when insights about the deceased’s life are revealed, for example, their suicidal thoughts and actions, through having access to their medical records. Here, a father describes reading his son’s records:

So, I imagine, we [healthcare] cannot take it. Now have we been doing this for a long time and we have come to the end of the road. And this guy [therapists] who spoke to my son, he even wrote, ‘I’m not coming any longer. I have been doing this for a very long time and I’m not getting anywhere’. But then you have to change to another path for fuck’s sake, and do something else! No, they don’t change to something else just business as usual, in the same shit.

A burdensome feeling is an increased sense of responsibility, involving many practical tasks that cannot be escaped. The responsibility implies a loss of energy, where the ability, for example, to receive news or receive visitors is reduced and the space for making new impressions is exceedingly limited. Despite this, the survivors are met by incomprehensible demands from those around them to move on after what has happened. After a long period of mental illness of the deceased, it can feel as though a “wet oppressive cloth” has been lifted, bringing a need to manage ambivalence, and a feeling of lightness and wellbeing that is at the same time filled with guilt. Impaired memory, increased vulnerability to stress, and difficulty sleeping are described to be caused by the death. Furthermore, survivors experience a lack of energy, a struggle against an uncompassionate employer, and guilt for what one’s children have to deal with. To feel some form of normalization, some survivors choose to move house and start over to avoid being constantly reminded of what has happened. The boundary between life and death is blurred, with a feeling of indifference to death, sometimes bringing with it one’s own suicidal thoughts and sometimes a newfound joy in life. The experiences of the loss provide insights into what life can be and reminds us of the vulnerabilities of life and what life can do to one’s own existence.

### The need to understand

After the death notification, a prolonged and energy-intensive hard effort begins, to try to understand the inconceivable. Gathering all the pieces becomes crucial in having the opportunity to solve the puzzle. The survivors are active and seek facts and information about the deceased. They receive information about the deceased from friends and other acquaintances, and by obtaining the deceased’s medical records and notes. Reading through the documents involves exposure to further painful insight about the deceased’s unknown life and their relationships with others become apparent. Sometimes, survivors are looking back many years, in some cases all the way back to childhood. The survivors, in solving their puzzle, turn to professionals in the form of seeking conversational support. This is something that the survivors arrange for themselves. The survivors spend a lot of time assembling the information and putting the pieces together. Puzzling, thinking, and trying to understand also implies experiencing an examination process similar to being the accused in a judicial trial.

It is a trial all the time (pause) Yes, it is a trial in my head all the time when you like (pause) to (pause) and I can (pause) I can get it to that is my fault. But then I know reason-wise I am not.

Through a process of self-examination, questions are asked about what should have been done and what should have been observed. At the same time, there are feelings of guilt. It is described as being placed in a yoke and the search sometimes reveals insights that ease off the yoke—easing one’s own guilt. At the same time, it is difficult to reach an end to this work. Gradually, an insight also emerges about the suicidal signals that existed before the suicide which were not understood then; these insights need to be managed. Below is a description of the moment when a picture of the puzzle begins to emerge:

Maybe two months before [the death] and all of a sudden we went down to her storeroom for a look. And, as I said, she was very orderly, you know, and she was down there and looked so – she could barely walk – and she said, ‘Oh, [participant’s name], look how many cartons and how much stuff there is.’ ‘But you’re kidding,’ I said, ‘There are some boxes on the shelves and they are marked and everything is folded and nice, this is like, this will take half an hour to clear out,’ I said, and then I thought, then, like all that was done back then. That like, yes, even then she had cleaned up like [you would] after a death long before that.

There is also a need to see the deceased in the morgue or in the place where the family member died. When it was not possible to see the deceased at the morgue, this is described as being a barrier to understand. Solving the puzzle and gaining an understanding creates a sense of peace and puts the survivor’s mind at rest once they stop dwelling on the matter, which also means avoiding questioning oneself:

You have to, no matter how much we try to dig into these things, we still do not know everything And then you have to realize that, yes, but here now okay, now I know enough.

### The ambiguous silence

The loss implies a clear need to share thoughts and feelings, by writing to or telling someone who genuinely and compassionately dares to listen sensitively and has the ability to remain quiet during the narration. For the survivor, it is a relief to be able to tell their story:

Then it was just, you weave it (pause) it just came like that. But then he just sat there and listened to my story. I would not say that it was hard to tell (pause) it is like, you unload emotional discharge a little bit, it is when you tell the story.

Close relationships undergo extreme challenges. Huge demands are placed on the family to cope with providing support to each other. For some of the survivors, they are in mourning themselves, and, at the same time, they need to be available to provide support for other family members. The foundations of support within the family are unstable, as the loss is managed differently by the family members, depending on their relationship with the deceased. Sometimes the closeness to other family members is soothing and partially supportive. This can also be described as strengthening the family relationships, for example, meeting as a family more often, and prioritizing the family more than before and taking financial responsibility for the children who remain. The survivors experience that they are self-limited in the meeting with others and at the same time have a major need to share their thoughts with someone outside the family. However, sharing thoughts with someone outside the family can also bring distress. One aspect of meeting someone outside the family with the similar situation is that it can be too much:

It is still difficult. I do think that the group meetings with SPES [suicide survivor organization] were good, but at the same time I felt sometimes that they gave me more pain rather that it did me good. Because you have your own grief and then you’re sitting with others with their grief and are listening to their stories.

The survivors have a need for practical help from those around them. Such practical help from the employer consists of, for example, reducing their requirements and allowing the survivors to attend their workplaces without heavy demands, or that they are offered conversational support from the occupational healthcare service. The practical help received from family members and people around them is a need for an everyday life that is familiar and a need for routines, and such support is also experienced as being listened to and encouraged.

The survivor has a need for flexible support, which means being offered continued support and being given a feeling of security by those who dare to listen. The survivors, on the one hand, sometimes lacked support from those where the support had been expected, which would lead to an unexpected exclusion of former friends. On the other hand, they are sometimes met with unexpected support from people who they thought were unlikely to do so. The silence from the healthcare service brings with it a feeling of being forgotten and abandoned, where they themselves must be active in seeking and receiving help.

Where can you call? We called the hospital / … / and we could come down to the hospital to some unit somewhere. But it all ended up in that we could not go to the hospital because he had not died in the hospital or something like that. Yes, it was some weird explanation like that.

There is also a great need to be treated with compassion and support, particularly when encounters with the healthcare service, other professionals, society, and other individuals feel unengaged, distanced, and cold. Even people who know the survivors seem to avoid any contact and even intentionally turn away. The survivor encounters inhumanity when society does not feel present and supportive.

Initially, a feeling of support is experienced in connection with the death notification being delivered, which, after a while, transforms into a lack of support. This sense of abandonment feels especially clear when promised support turns out to be non-existent:

The police officer went / … / and they [police officers] said: ‘You know where we are,’ and they also said that they work until then and then. They work until nine or something like that. So I got a telephone number and then a name I think, on a piece of paper. / … / But at least I got this person on the line finally: ‘We need help’ ‘Yes, no but, but we cannot come we do not have any help to give. No, we cannot send anyone but we would like that one of children come to us so we can swab them’ [take saliva samples].

The genuine compassion when the death notification is delivered confirms and responds attentively to the need for calm, closeness, honesty, and touch. It occurs when the person who delivers it introduces themselves, maintains a sense of calm, promotes a feeling of care, is kind, and is prepared to listen. A description of how compassion can also be conferred in a phone call is made here:

Then she [police officer] stayed calm all the time. You had the feeling that she cared about me in some way and that she wanted to frame the conversation in a sort of security in some way, in the middle of all the strange things that of course happened then. And then you also got the feeling of care when I started, like, ‘Yep, but what will happen then? And will we find out about it? And do we expect to get anything home, or?’ No, we do not. ‘But I [police officer] can call you later.’ In this way you felt that she wanted it to be good. And you feel like she took care of you in the way that she could on the telephone. And she stayed calm, collected, and pleasant, and yeah, that conversation felt very good.

In contacting and in meeting with the healthcare service after the death, the survivors describe an inability from the healthcare professionals to show compassion. Even a lack of compassion in those delivering the death notification was described:

Maybe they [police officers] said ‘Bye’. I do not know. Even if I had not been able to say anything, just ‘Yep’. Or something. I think that it (pause) I do not know. A certain compassion was missing. Because you are in shock. They [police officers] come and give you a shock. And then you can just say, ‘Bye, we’re leaving now’. Then you know that here is someone who, they have just announced, but they have also cared I do not have any memory of when the police officers actually went, it is that. I see it very clearly now, that they would have said ‘Bye’. Maybe only ‘Bye’. Perhaps, ‘Do you want to ask something?’ Of course, you cannot when you are in shock, but they could still ask the question. Because inside me I have registered it for maybe a long time afterwards.

### Memories as an eternal companion

There is a need for sharing memories with family, friends, and others about the deceased, particularly when others choose not to talk about the family member. This silence is experienced as if the family member never existed and intensifies the feelings of emptiness.

No one even mentions him, of those who have found out [that he died by suicide] / … / but I do not want them to remove him. He should be allowed to join. I wish they might just [ask] ‘How was (the name of the deceased) when he was ?’ / … / I would like it to be a bit like that, so that he can be a bit alive.

There is a value in preserving the memory and in which way the survivors accomplish this is varied. Some have the need for solitude and having an opportunity to meet the deceased in a secluded “room” and to preserve there the memory of the family member. Memories of the deceased reveals itself in both the good times and the bad. The memory can also bring to life without being an active prompt and can come at any time—memories that need to be managed and, in some cases, even processed. Further, with an emptiness and the need to adjust to a life where the family will never be the family that it once was. Grief is consciously managed as a way to prevent a total obliteration of the deceased’s memory and imprint. This conscious management becomes a constant companion in life:

My mom and dad have passed away, but still it’s like not the same. This was like (pause) losing an arm or a part of the body. Yeah. And now I am going to learn how to live without a part of my body, in a different way. In the memory somehow. Mm (pause) yeah, but it is like I have been amputated somehow. He amputated me.

### The fear of being convicted

There are barriers to talking about one’s own feelings and it is not possible to unburden one’s feelings without affecting others. In some situations, survivors choose not to discuss their emotions with other, to avoid affecting the atmosphere. Sometimes they choose not to tell others about the real cause of death to spare themselves from being blamed by others, and other family members from feeling guilty.

These experiences are like secrets that are carried through barbed wire and that are only revealed after the wire has torn open wounds, such as in sudden and unexpected moments, when the survivor is forced to explain why the family member is not with us any longer. There is a constant fear of being judged by others and being blamed, or that the whole family is being judged in the eyes of others and being held responsible for what has happened. The conscious choice to tell one’s story is made only when trust is established. Often, in meetings with other suicide survivors, the survivors are seen as being on the same level and, for that reason, such silences can be supportive and not judgemental.

You do not have explain so much, what you feel and how you feel. And it is about the silence, the silent language, you know exactly how it is. No one asks the extra question or thinks that it is odd, what you said or that. You understand each other exactly.

It feels like being in a vacuum, not being able to share thoughts with others and about the difficulties that come with the fear of being judged. Reluctantly, the need to be seen is weighed against the risk of being attributed an undesirable identity; to be seen as a family where someone had died as a result of suicide:

I feel that there will be (pause) It is something that you defend yourself against hearing. And it is very hard to hear that a young person committed suicide or ‘What is going on?’ And that also creates a lot of fantasies about what our family really looked like.

## Discussion

Based on the phenomenon of losing a family member by suicide and the present results, the question of “losing” should be understood in relation to “being” a suicide survivor, that is to say, human existence in all its dimensions. The opening question of the interviews was directed at the experience of losing, while the answers were often about being, which implies a tightly entangled meaning that is impossible to separate. The survivors did not only describe losing a family member, they also described a need to orient themselves within the lifeworld of other human beings. This could explain the closely intertwined and dialectical relationship between being and losing, which clarifies “being into the world” for the survivors. Being is always about “being into the world”, and the world is always something that we share with others (Heidegger, 1927/1998; Merleau-Ponty, 1945/2002, 1948/1968). In this study, being a suicide survivor signifies existential loneliness and life suffering.

The results indicate that survivors seek a deeper understand by solving a puzzle in order to ease off the yoke and cope with the question of guilt. Previous studies have described that survivors struggle in silence with unanswered questions (Bowden, 2017), searching for reasons why the suicide happened (Begley & Quayle, 2007), and needing answers to central questions such as, “*Why did he commit suicide*?” (Fielden, 2003). It seems to be central for the survivors to understand what, in some sense, is elusive and not fully possible to understand. An increased understand does not necessarily reduce feelings of guilt, but instead facilitates the understand that the survivors can cope with the feelings of guilt in everyday life. It is not the answers to the questions “Why?” that are important *per se*, instead, it is vital to really understand.

The lived experiences of the survivors in the present study indicate a profound life suffering and feelings of involuntary loneliness. The survivors described a continual fear of being judged. Previous studies also describe that survivors blamed and judged themselves for the death (Peters et al., 2016a), felt blame and judgement from others (Ford, 2016; Sheehan et al., 2018), and felt judged by the first responders in the immediate aftermath of a suicide (McKinnon & Chonody, 2014). By condemning another human being and acting as a judge, much suffering is caused by feelings of being accused, blamed, or judged by others. The suffering, guilt, and pain may be found among family members who occupied a close relationship with the deceased. The feeling of humiliation and shame is a type of torment that afflicts a person who fights against suffering (Eriksson, 2006).

The survivors experienced barriers to talking about one’s own feelings and burdens, and it is impossible to ease these without affecting others. A previous study has described similar results, where survivors felt a sense of responsibility to alleviate the discomfort demonstrated by others (Peters et al., 2016a). The survivors in the present study needed to share their thoughts and feelings with others, and sometimes the expected support was lacking. The lack of support also meant a lack of opportunities to talk about and maintain the memory of the deceased. Similar results have been found in a phenomenological study, where the survivors were found to have kept the memory alive through themselves and by regarding the deceased as still being a part of the family, and never saying goodbye (Kinsey, 2019). In a study of the enigmatic phenomenon of loneliness, the meaning of loneliness can be understood as an existential deficit and as being rejected by people who they want to be with, which makes this imposed loneliness painful (Dahlberg, 2007). Further, the suffering caused by silence from the people around them left the survivors in a state of involuntary loneliness. An additional suffering in care and healthcare was caused by a violation of the person’s dignity through omitted care or a non-caring attitude. According to Eriksson (2006), suffering in care and healthcare often arises from unconsciously acting, deficient knowledge, and a lack of reflection. Other studies have also expressed deficient availability and compassion in healthcare organization (Peters et al., 2016b), which can also be considered as suffering caused by healthcare. Instead, healthcare professionals need to support and provide compassionate care to the survivors. Compassionate care is, according to Dewar et al. (2014), a relational activity involving noticing another person’s vulnerability, experiencing an emotional reaction to this, and acting in some way. In the present results, it was obvious that the usual support mechanisms within the family became unstable as the survivors coped with the loss differently, depending on their relation to the deceased family member. Previous studies describe similar results, for example, that the suicide leads to the fracturing of family relationships (Peters et al., 2016a), strained family relationships as family members are unable to acknowledge the grief of others in the family (Tzeng et al., 2010), avoidance and distancing from close relationships, superficial or troubled close relationships (Hoffmann et al., 2010), and family conflicts (Lee et al., 2017). Further, the present study indicates that the support from others is not taken for granted. Sometimes the survivors encountered incomprehensible demands from people around them to move on with their lives. Previous studies also describe that survivors were confronted with avoidant and even repellent behaviours from others, instead of receiving support (Peters et al., 2016a), making unreasonable demands on the survivor with statements such as, “let it go and move on” (Kinsey, 2019; Sheehan et al., 2018).

The survivors concealed the loss and carried the loss like a secret, as partly revealed in this present study, and as also described in other studies (Azorina et al., 2019; Peters et al., 2016a). In a conceptual review of loneliness, existential loneliness was found to offer a different perspective on loneliness when compared to social or emotional loneliness. Existential loneliness means not simply the absence of meaningful relationships, it also signifies a feeling of fundamental separateness from others and the wider world, often in conjunction with traumatic events and death (Mansfield et al., 2019). The deepest feeling of loneliness occurs perhaps when a person is not seen by others, which is perhaps the deepest suffering of all. Not being seen is, therefore, in some sense, like being considered as being “dead” (Eriksson, 2006).

### Methodological considerations

This topic may be difficult to talk about and, with respect for the complexities of the phenomenon, the researchers chose the RLR approach for this study in seeking the meanings of a phenomenon (Dahlberg et al., 2008). A strength is that both women and men were included in this study; as Maple et al. (2014) point out, the existing literature lacks studies about men’s experiences as suicide survivors, along with experiences of losing a woman by suicide. A further strength is that the survivors had a variety of relationships with the deceased, which is also an element that is missing in previous studies (Sveen & Walby, 2008). It can also be considered a strength that the participants were not recruited from support groups, unlike most studies conducted to date (Maple et al., 2014). The demographic variation among the participants is judged to also have contributed important and varying experiences (Dahlberg et al., 2008).

The study has some limitations regarding the risks associated with recruiting a selective sample. First, most of the participants had received the death notification from police officers. Despite this, the results might be transferable to other professionals and also to everyone who may encounter suicide survivors. Second, no young adults participated in the study. It is conceivable that, if the advertisement had been posted on other platforms, perhaps we may have reached them. This raises the likelihood that certain nuances of the phenomenon were missed, but the essential structure of meanings would probably remain the same.

Promoting additional objectivity is the rationale for posing a wide opening question, so the researchers do not tend to steer the conversations with participants in any one direction except towards talking about the phenomenon. The open question allows them to feel free to choose where to begin. In that way, the participants had control of the interviews and the first author could adapt to the participant. This also minimizes the risk of steering them towards a specific answer and allows the participants to speak about as much of the phenomenon that they wish to and not only about the things that the researcher finds interesting. It is also a way to ensure that the pre-understanding of the researcher does not dominate the interview (Kvale & Brinkmann, 2009). The decision to perform a second interview was a good one, as it gave the participants an opportunity to clarify and provide more nuanced experiences. Having a difficult story to tell acted as a release for them, and a re-interview allowed the interviewer to focus more on asking them to describe their impressions of the phenomenon. All participants said before the first interview was finished, “*Can we meet again? I have probably missed something*”. However, the researcher must keep in mind that they have met before and use openness and curiosity to look for new insight and otherness, especially during the second interview. These are particularly strong emotional stories, and, to find a balance between fellow human being and a researcher, the interviewer had to focus on the phenomenon instead of being drawn into the story and completely lose the phenomenon. According to Dahlberg et al. (2008), openness, curiosity and adopting a bridling attitude helps the interviewer to perform these emotionally strong interviews to reveal rich meanings by moving between subjectivity and objectivity. Further, to promote the opportunity to find the essence of a phenomenon, the data have to be rich in meanings (Dahlberg, 2006). In this study, the interviews with the survivors were very rich.

On one occasion, it was necessary for the first author to change from the role of researcher to the role of prehospital emergency care nurse to make an assessment about a participant’s disclosure of suicidal thoughts.

## Conclusions and implications

The loss of a family member by suicide is a phenomenon characterized by additional burdens and suffering being placed on those close to the deceased. Being a survivor implies existential loneliness and life suffering, which are new insights revealed in this study. Searching to understand a constant fear of being judged, and being attributed an undesirable identity, are partly new insights. The silence from people around the survivor, a welcome need to speak to someone, and the struggle to preserve the memory and imprint of the deceased, are found in other studies and confirmed in the results.

Because the survivors are extremely sensitive to meeting others, it seems important that professionals are aware of this sensitivity, which means that there is an extra need for them to be attentive and responsive in their relationship with the survivor. From the very first stage, in connection with the death notification, professionals should already accept survivors as suffering human beings and the healthcare professionals should consider them as patients. Hence, the healthcare professionals should take the initiative and provide compassionate care. Such support might reduce life suffering, counteract stigma and involuntary loneliness, and work simultaneously as suicide prevention.
